# Mid-urethral sling with proper sling tension is an effective treatment for stress urinary incontinence in women after pelvic radiotherapy: a pilot study of case series

**DOI:** 10.3389/fsurg.2024.1475030

**Published:** 2025-01-03

**Authors:** Xing Guan, Fei Wang, Di Zhang, Peng Qiao, Yan Qin, Biao Wang

**Affiliations:** ^1^ Department of Urology, Beijing Chao-Yang Hospital, Capital Medical University, Beijing, China; ^2^ Department of Urology, Guangxi Hospital Division of The First Affiliated Hospital, Sun Yat-sen University, Nanning, Guangxi, China

**Keywords:** stress urinary incontinence, pelvic radiotherapy, mid-urethral sling, lower urinary tract symptoms, pelvic tumor

## Abstract

**Background:**

At present, consensus on the management of female stress urinary incontinence (SUI) after pelvic radiotherapy is lacking. We aim to assess the clinical effects of mid-urethral sling (MUS) for the treatment of SUI after pelvic radiotherapy in women.

**Methods:**

We conducted a retrospective review of the clinical database of female with SUI after pelvic radiotherapy from June 2015 to February 2022. The clinical efficacy was evaluated by International Consultation on Incontinence Questionnaire-Short Form (ICI-Q-SF) questionnaire, maximum flow rate (Qmax) and postvoid residual (PVR) urine. All patients were reviewed postoperatively in an outpatient clinic.

**Results:**

We identified 26 patients with mean age of 59.35 ± 7.32 years. All the patients who suffered from SUI had a history of gynaecological malignancies and received pelvic radiotherapy. 21 patients (80.77%, 95% CI: 0.621–0.915) were considered to have successfully improved after surgery, the ICI-Q-SF scores were lower than the pre-operative at 2 weeks, 6 months and 1 year postoperatively (*P* < 0.01). After 1-year follow-up, none of the patients had mesh erosion.

**Conclusion:**

SUI following radiotherapy for the treatment of pelvic malignancy can be challenging to manage. MUS is a highly effective and safe option for the treatment of SUI after radiotherapy, additionally, that proper sling tension is the key to the success of the procedure.

## Introduction

According to previous studies, approximately 40% of patients with genital, urological and low gastrointestinal tumors have received pelvic radiotherapy (1). Although radiotherapy can destroy cancer cells and inhibit their spread (2, 3), it also triggers fibroblast senescence, affects mesenchymal cells differentiation, and even enhances colloid cells replication (4, 5), leading to changes in tissue stiffness and elasticity (6, 7). Consequently, radiotherapy for pelvic tumors can induce alterations in the pelvic floor structures, resulting in stress urinary incontinence (SUI). Due to the detrimental impact of radiotherapy on the management of SUI, numerous clinical trials of mid-urethral sling (MUS) have excluded individuals who have undergone pelvic radiotherapy from the study (8).

With the development of anti-incontinence surgery, MUS had gradually become the first choice for treatment of SUI (9). The treatment of gynaecological malignant tumors (e.g., hysterectomy and radiotherapy) can cause SUI. Of these, cervical and endometrial cancers require radical hysterectomy, and some patients choose radiotherapy as further treatment. The incidence of SUI is approximately 24%–29% in patients with cervical cancer (10), whereas it is 46.8% in patients with cervical cancer after hysterectomy (11). The incidence of urinary incontinence was higher in patients who received radiotherapy after hysterectomy compared to patients who only underwent hysterectomy (12).

The treatment of pelvic malignancies may result in dysfunction of urinary storage and urination. A study revealed that hysterectomy and radiotherapy affects the innervation of bladder, which leads to 9 bladder contractile dysfunction (13). Although there are few studies on urodynamic features after pelvic radiotherapy, we note that 15%–20% of patients in these studies developed detrusor instability and urinary frequency, accompanied by decreased bladder compliance and bladder overactivity after treatment (14–16). Meanwhile, compared to hysterectomy alone, radiotherapy can cause a decrease in pelvic floor muscles contractility (17). In a study of the effects of radiation and chemotherapy for cervical cancer on pelvic floor muscle function, it was found that radiation and chemotherapy resulted in pelvic floor dysfunction, especially at the end of treatment (18). Bladder dysfunction during storage and voiding after radical hysterectomy and radiation therapy often co-occurs with SUI, resulting in complicated conditions. Moreover, the fibrosis of pelvic floor tissue caused by radiotherapy can lead to more severe urinary incontinence. Currently, there is no consensus on the treatment of urinary incontinence after radiotherapy, thereby the treatments of physicians vary.

Despite the substantial burden of disease, only a limited number of researchers have focused on characterizing SUI following radiotherapy for female pelvic malignancies. MUS surgery has emerged as a widely favored and effective treatment for female SUI. In one research, MUS was administered to patients who had undergone radiotherapy and radical hysterectomy, which found that the recurrence rate of stress incontinence was 100% (19). Conventional sling procedures often struggle to control leakage effectively under tension-free conditions, primarily due to tissue stiffness following radiotherapy. Moreover, there is a notable absence of consensus regarding the management of female SUI after pelvic radiotherapy. Regarding this dilemma, we report our experience in managing patients who have undergone MUS operation for SUI post-radiotherapy, delineate the characteristics of SUI after pelvic radiotherapy, and present the results of this surgical intervention.

## Materials and methods

### Study population

This retrospective study focused on patients diagnosed with SUI subsequent to undergoing radiotherapy for pelvic tumors and who underwent MUS procedures at our institution between June 2015 and February 2022. The diagnosis was based on comprehensive history, clinical presentation, physical examination, uroflowmetry, PVR measurement and urodynamic testing. Data extracted included patient demographics, medical history, severity of SUI and any associated postoperative complications, and urogenital symptoms.

Patients were included in the study if they met the inclusion criteria: (1) Female patients >18 years of age. (2) Patients diagnosed with pelvic malignancy and treated with radiotherapy. (3) Patients treated with MUS. Patients with other urinary diseases (e.g., bladder neck obstruction, urethral stricture, bladder prolapse), who had undergone operation for urinary incontinence, or who were unable to complete follow-up were excluded from the study. The protocol was reviewed and approved by the institutional review committee of Beijing Chao-Yang Hospital (approval number: PX2020015) and informed consent was taken from all individual participants.

### Procedure management

The surgical procedures were generally carried out under general anesthesia. All procedures were performed by the same surgical team.

The patient was initially positioned in the gynecological posture. The operative area was prepped with a standard antiseptic solution and covered with multiple drapes. An 18 Fr Foley catheter was inserted to empty the bladder. Labia minor was suspended by fixation to the skin with nylon sutures a few centimeters above the vulvar ostium, inside the thigh folds, in order to expose the vulvar vestibulum. Inject 20 ml of saline into the vagina and urethral space. A median sagittal incision of the vaginal wall was started at this level and was continued proximally (towards the vaginal pouches) over a 1 cm distance, both vaginal mucosal and sub-mucosal tissues were incised. Minimal para-urethral sub-vaginal dissection was then carried out laterally with the scissors, over a few millimeters distance, on either side. After the dissection pathways were successfully established, the introducer was advanced towards the retropubic space. The bladder was filled with 300 ml of saline and then underwent an intraoperative cystoscopy to check for the presence of bladder injury. Subsequently, The abdominal compression test was performed with a bladder volume of 300 ml, aiming to adjust the tape to enable a drop of saline to escape from the outer meatus of the urethra upon strong abdominal compression. For procedure, the surgeons were instructed to place the sling with appropriate tension rather than “tension-free”. The tape ends were cut in the subcutaneous layer and the incisions were closed. Finally, the vaginal incision was closed with absorbable sutures.

### Main study outcomes and follow-up evaluation

The primary endpoint was the ICI-Q-SF questionnaire for leakage symptom, and the secondary endpoints included uroflowmetry and postoperative complications such as dysuria and sling exposure. The postoperative objective success was defined as no urine leakage during a cough stress test or answering “no” to the ICIQ FLUTS question: “does urine leak when you are physically active, exert yourself, cough or sneeze?” (20, 21). Patients were discharged on criteria of improving urinary leakage symptom as well as Qmax > 15 ml/s or PVR < 50 ml. Patients who meet the criteria can be discharged. Patients underwent a review at 2 weeks post-surgery, where uroflowmetry and PVR were conducted at the outpatient clinic. Urinary symptoms were evaluated using the ICI-Q-SF questionnaire. Subsequently, all patients were followed up via telephone at 3, 6, and 12 months postoperatively. During follow-up period, ICI-Q-SF questionnaire, uroflowmetry, PVR, and any postoperative complications were recorded. Pre- and post-operative data of ICI-Q-SF scores, Qmax and PVR were compared for each patient.

### Statistical analysis

The results were presented primarily using descriptive statistics due to the relatively small sample size. Where appropriate, continuous variables with normal distribution were presented as means ± standard deviation (SD) and compared by Paired *t*-test. While continuous variables with non-normal distribution were reported as median with interquartile range (*P25*, *P75*) and compared by Wilcoxon signed-rank test. All tests were two-sided with *p* value < 0.05 to be considered statistically significant. All statistical analyses were performed using SPSS statistical software version 26.0 (IBM, Chicago, IL, USA).

## Results

A total of 26 patients with objective evidence of SUI after radiotherapy for pelvic tumors were enrolled in our study. The mean age and body mass index were 59.35 ± 7.32 (range 43–70) years and 25.72 (22.79, 27.86) kg/m^2^, respectively. The baseline characteristics of patients, the history of pelvic tumors, and preoperative parameters of uroflowmetry were demonstrated in Table 1.

**Table 1:** Patient demographics and clinical features.

Characteristics	All patients (*n* = 26)
Age (years)	59.35 ± 7.32
BMI (kg/m^2^)	25.72 (22.79, 27.86)
Duration of tumor (years)	5.00 (4.00, 7.00)
Primary tumor, *n* (%)	
CC	19 (73.08%)
EC	7 (26.92%)
Radiotherapy times	20.00 (15.75, 25.25)
Total radiation dose (Gy)	37.50 (30.00, 46.25)
Abdominal pressure voiding, *n* (%)	
Yes	2 (7.69%)
No	24 (92.31%)
VLPP (cmH_2_O)	57.69 ± 23.72
Qmax, (ml/s)	26.40 (22.78, 43.73)
PVR (ml)	0.00 (0.00, 0.00)

BMI, body mass index; CC, cervical cancer; EC, endometrial cancer; VLPP, valsalva leakage pressure point; Qmax, maximum uroflow rate; PVR, postvoid residual urine volume.

All 26 patients completed the surgery successfully. The comparison of Qmax and PVR at 2 weeks postoperative and preoperative was listed in Table 2. Postoperatively, the symptoms were improved and ICI-Q-SF was decreased significantly compared with the preoperative. As shown in Table 3, the ICIQ-SF scores were lower than the pre-operative at 2 weeks, 6 months and 1 year postoperatively, and the difference was statistically significant (*p* < 0.01).

**Table 2:** Comparison of Qmax and PVR at 2 weeks postoperative and preoperative.

Parameter	Preoperative	95% CI	Postoperative	95% CI	*d*	*p* value
Qmax (ml/s)	26.40 (22.78, 43.73)	26.68–38.11	22.35 (13.08, 27.83)	17.58–25.86	0.703	0.001
PVR (ml)	0.00 (0.00, 0.00)	−2.43–9.74	0.00 (0.00, 65.00)	9.46–162.07	0.613	0.016

Qmax, maximum uroflow rate; PVR, postvoid residual urine volume; CI, Confidence interval; *d*, Cohen's d. Data were presented as *M* (*P25, P75*) and compared by Wilcoxon Signed Ranks Test.

**Table 3:** Comparison of ICI-Q-SF scores before operation and 2 weeks, 6 months and 1 year after operation.

Items	Preoperative	95% CI	2 weeks	95% CI	*d*	*p* value	6 months	95% CI	*d*	*p* value	1 year	95% CI	*d*	*p* value
Frequency or UI	4.00 (4.00, 5.00)	4.13–4.64	2.00 (1.00, 2.00)	1.18–2.12	2.921	<0.01	1.00 (0.00, 2.00)	0.68–1.70	3.178	<0.01	1.00 (0.00, 1.25)	0.58–1.66	3.129	<0.01
Amount of leakage	6.00 (4.00, 6.00)	4.74–5.80	2.00 (2.00, 2.00)	1.59–2.56	2.539	<0.01	2.00 (0.00, 2.00)	1.01–2.06	2.858	<0.01	2.00 (0.00, 2.00)	0.88–2.05	2.757	<0.01
Overall impact of UI	10.00 (10.00, 10.00)	9.27–9.96	2.00 (0.75, 4.00)	1.80–3.82	2.599	<0.01	2.00 (0.00, 3.00)	1.01–2.99	4.156	<0.01	1.50 (0.00, 2.25)	0.86–2.98	3.953	<0.01
Sum scores	20.50 (18.00, 21.00)	18.31–20.31	6.00 (4.00, 9.25)	4.78–8.30	3.600	<0.01	5.00 (0.00, 6.00)	2.79–6.67	3.816	<0.01	4.50 (0.00, 5.75)	2.39–6.61	3.624	<0.01

ICI-Q-SF, International Consultation on Incontinence Questionnaire-Short Form; UI, urinary incontinence; CI, confidence interval; *d*, Cohen's d. Data were presented as *M* (*P25, P75*) and compared by Wilcoxon Signed Ranks Test.

During our follow-up, 21 patients (80.77%, 95% CI: 0.621–0.915) were considered to have successfully improved after surgery. 5 patients (19.23%, 95% CI: 0.085–0.379) experienced dysuria after surgery, case 3 had no voiding trouble on postoperative day 1 and met the discharge criteria. At the first review 2 weeks after surgery, her Qmax was 3 ml/s and PVR was 700 ml, the patient was performed urethral dilatation at the outpatient clinic, and the patient had significant amelioration of symptoms after urethral dilatation with Qmax increasing from 3 ml/s to 15.7 ml/s and PVR decreasing from 700 ml to 0 ml. Case 5 experienced dysuria after 2 weeks postoperatively, the patient received multiple urethral dilatation with Qmax varying from 12 ml/s to 12.1 ml/s and PVR varying from 250 ml to 240 ml. There was no significant alleviation of dysuria symptoms at 3 months postoperatively, and the patient underwent sling release at 6 months post-surgery. After sling release, dysuria was relieved and the problem of urinary incontinence was well controlled. Moreover, the PVR of case 11, case 18, and case 24 at 2-week postoperative were 150 ml, 670 ml, and 200 ml, respectively. The patients were treated with urethral dilation, and their symptoms of dysuria were relieved with PVR of 0 ml. After 1-year follow-up, none of the patients had sling exposure.

## Discussion

The understanding of the presentation, diagnosis, and treatment of SUI has developed over the last 20 years. However, there is still no consensus on the treatment options and efficacy for urinary incontinence occurring in patients with cervical cancer, and the outcome may be affected by the primary disease. Pelvic floor muscle training (PFMT) is available as an initial therapeutic option; unfortunately, up to now, there is only limited clinical data on its effects (22). Periurethral or transurethral injection of bulking agents is currently the only treatment modality with research evidence to show its effectiveness for urinary incontinence after radiotherapy (23–25). Since the long-term effects of radiotherapy on the voiding function of the lower urinary tract are commonly irreversible and progressive, the manifestations of urinary incontinence post-radiotherapy exhibit variability. Opting for an incorrect therapeutic strategy may lead to symptom recurrence and exacerbation.

In our study, all patients had undergone radiotherapy for gynecological malignancies and subsequently developed urinary incontinence. All patients underwent trans-retropubic vaginal tape, with follow-up assessments conducted at 2 weeks, 6 months, and 1 year after surgery. Based on the ICI-Q-SF scores, symptom improvement was observed at the 2-week postoperative mark. Notably, beyond 6 months postoperatively, the sling fused with surrounding tissue and the subjective symptoms of the patients improved more significantly. The ICI-Q-SF score decreased from 20.50 (18.00, 21.00) to 4.50 (0.00, 5.75), (*p*<0.01). In contrast to previous extensive case analyses where MUS was ineffective after radiotherapy (26), 21 of 26 patients in the present study experienced improvement in symptoms. During the 1-year follow-up after the operation, there were no complications such as sling erosion, with only 1 case undergoing sling release surgery due to dysuria. Consequently, the success rate of the procedure was 80.77% at 1-year follow-up.

Among the 26 patients in our study, 2 patients exhibited abdominal pressure voiding during the preoperative urodynamic evaluation. Since both surgical treatment and radiotherapy of gynaecological tumors can affect bladder innervation and damage the detrusor function (13, 15). Currently, there is a lack of research addressing whether retropubic slings with appropriate tension might exacerbate dysuria in such patients. Case 5 had to undergo sling release surgery because of postoperative dysuria and urinary retention. However, it is worth noting that although the PVR in case 1 was 110 ml at 2 weeks after the procedure, the patient did not undergo further treatment and was followed up regularly and had a PVR of 0 ml after 1 month postoperatively. We consider the increased residual urine volume in this patient to be associated with postoperative periurethral edema, and the dysuria was relieved after the edema subsided 1 month after surgery. Therefore, for patients with urinary incontinence after radiotherapy, if the function of detrusor has been impaired before surgery, it is necessary to adequately communicate with the patient before the operation that there may be aggravation of dysuria or even urinary retention after treatment.

All patients with urinary incontinence after radiotherapy should undergo urodynamic examination before surgical intervention to exclude overflow urinary incontinence. While “tension-free” is a crucial aspect of retropubic tension-free vaginal tape (TVT) surgery, in the context of pelvic floor tissue stiffness after radiotherapy, appropriate sling tension on urethra becomes essential to relieve urinary leakage symptoms. The balance between the control of urinary incontinence symptoms and postoperative dysuria is difficult to meet. For example, the sling tension of case 5 that can control incontinence symptoms means severe dysuria or even urinary retention after surgery, but in 21 patients did not suffer from dysuria while controlling the symptoms of urinary leakage. This proved that proper sling tension is the key to successful surgical treatment of MUS for SUI after pelvic radiotherapy. Unfortunately, we failed to propose a method to quantify sling tension, which will be the priority of our future research endeavours.

The postoperative complications of urinary incontinence after radiotherapy are mainly sling erosion, which is related to the poor ability of tissue self-repair after radiotherapy and may also be associated with adhesions of the pelvic floor surrounding tissues after radiotherapy (27, 28). For the 26 patients in this study, we preserved the thickness of the vaginal wall and periurethral tissues as much as possible during the procedure, which we considered to avoid the occurrence of sling exposure. After 1-year follow-up, none of the patients had sling exposure.

This present study is innovative because few literatures focus on patients with SUI to research the specificity of urinary incontinence after radiotherapy. There is no definitive evidence that any treatment method has a better effect on this disease. In the present study, we involved the largest number of cases in such research works. Despite this, the results of our statistical analysis are still limited due to the small number of cases and the short follow-up period. We will obtain more cases and longer follow-up results in future works.

## Conclusion

MUS with appropriate sling tension emerged as a simple, safe, and effective treatment for SUI, with the improvement of incontinence in 80.77% of patients. It could be the first choice for patients who presented SUI after pelvic radiotherapy, and long-term follow-up is mandatory for patients presenting with SUI after treatment of pelvic tumors, as well as the treatment should be individualized.

## Data Availability

The raw data supporting the conclusions of this article will be made available by the authors, without undue reservation.
